# CD44 as a Predictive Biomarker in Non-Small Cell Lung Cancer Patients Treated with Radiotherapy

**DOI:** 10.51894/001c.164021

**Published:** 2026-07-31

**Authors:** Mabintou Darboe, Chad H. Stone, Kenneth A. Jenrow, Karen Lapanowski, Mei Lu, Alexandra R. Sitarik, Lois E. Lamerato, Astha Dalal, Matthew J. Jones, Avielle Movsas, Hammoud T. Zane, Nancy Peshkin, Kathleen M. Roszka, Munther Ajlouni, Sanath Kumar, Indrin J. Chetty, Michael J. Simoff, Benjamin Movsas, Renate Parry, Stephen L. Brown

**Affiliations:** 1 College of Osteopathic Medicine, Michigan State University

## 16

### Introduction

Non-small cell lung cancer (NSCLC) remains a leading cause of cancer mortality, and outcomes after definitive radiotherapy for locally advanced disease are still poor. Predictive biomarkers that anticipate tumor response to radiotherapy are limited; CD44, a cancer stem cell or tumor-initiating cell marker linked to radioresistance, is a promising candidate.

### Objectives

To determine whether pretreatment tumor CD44 expression predicts local response of NSCLC to radiotherapy.

### Methods

Tumor specimens from NSCLC patients (n=133) treated with radiotherapy alone or combined radiochemotherapy were analyzed using a protein expression assay. Patients presented with stage I (21%), II (15%), or III (64%) disease. Local tumor control following radiation therapy was assessed, and logistic regression was used to evaluate factors predicting local failure, including CD44 expression.

### Results

CD44 expression exhibited both intra-tumoral and inter-tumoral heterogeneity. In univariate logistic regression, higher CD44 expression was associated with increased odds of local tumor control failure across intensity, proportion, and composite measures (*p* = 0.027, *p* = 0.014, and *p* = 0.012, respectively). Overall, higher CD44 expression was significantly associated with increased odds of local failure after radiotherapy (OR 1.44, 95% CI 1.08–1.92).

### Discussion

Pretreatment CD44 expression was associated with local control failure following radiotherapy in NSCLC. CD44 may serve as a predictive biomarker to identify patients at higher risk of local failure and inform treatment tailoring, but these findings require validation in larger, independent cohorts.

